# Supplementary material for: An in-situ polymerization strategy for gel polymer electrolyte Si||Ni-rich lithium-ion batteries
Source: Nat Commun. 2024 Jun 25;15:5375. doi: 10.1038/s41467-024-49713-z (PMC11199651; doi:10.1038/s41467-024-49713-z)
Supplement: Supplementary file 1 — Supplementary Information [file 41467_2024_49713_MOESM1_ESM.pdf]

Supplementary Information

An in-situ polymerization strategy for gel polymer electrolyte Si||Ni-rich lithium-ion batteries

Miao Bai[1], Xiaoyu Tang[1], Min Zhang[1], Helin Wang[1], Zhiqiao Wang[1], Ahu Shao[1], Yue Ma*[1]

[1]State Key Laboratory of Solidification Processing, Center for Nano Energy Materials, School of Materials Science and Engineering, Northwestern Polytechnical University, Xi'an 710072, P. R. China.

Corresponding author: mayue04@nwpu.edu.cn (Y. Ma)

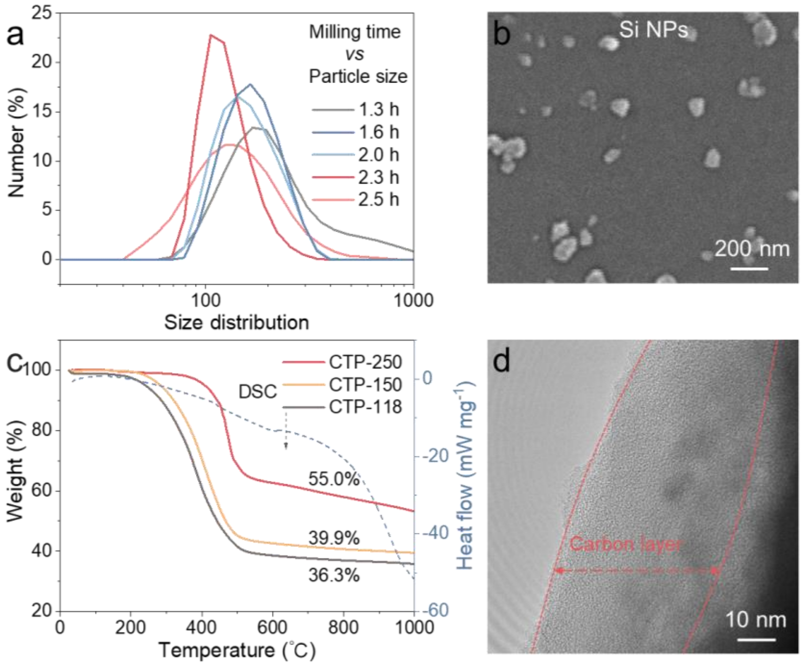

Fig. S1 (a) PSD curves of Si NPs upon the sand-milling process from 1.3 h ~ 2.5 h. (b) FESEM image of Si NPs after 2.5 h sand-milling process. (c) TG spectra of CTP with different softening point. (d) HRTEM image of the carbon layer of the Si/C-5 composite.

Notably, the CTP with high softening point (250 °C) was chosen, which possess adequate toluene insoluble (TI), quinoline insoluble (QI) contents and less volatile species (Fig. S1c).[69]

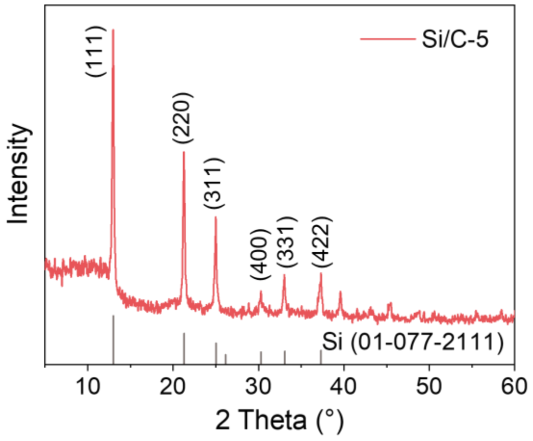

Fig. S2 XRD patterns of Si/C-5 composite.

Fig. S2 shows the XRD patterns of Si/C-5 composite. It can be easily concluded that diffraction peaks can be well indexed to the (111), (220) and (311) planes of cubic Si structure (ICSD number 01-077-2111).

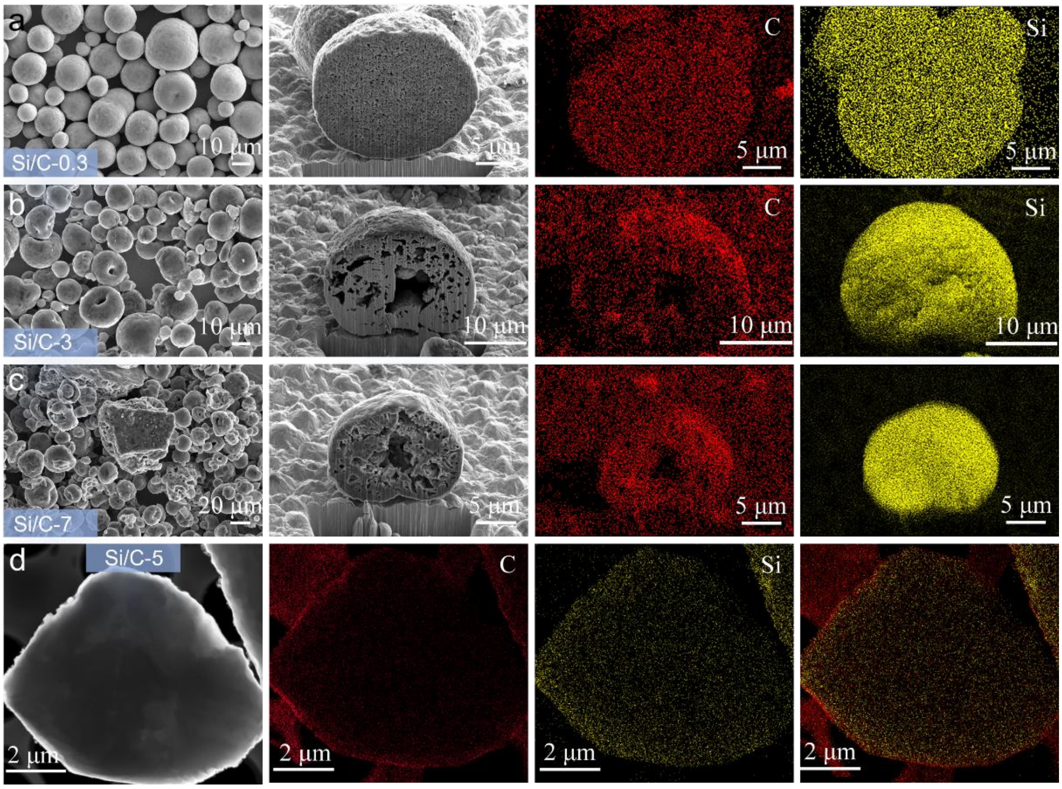

Fig. S3 The cross-sectional image of the (a) Si/C-0.3, (b) Si/C-3, and (c) Si/C-7 composites and corresponding EDS elemental maps of C and Si. (d) TEM image of the Si/C-5 composite and corresponding EDS elemental maps of C and Si.

It is noteworthy that Si/C-7 with 7 μm CTP exhibit the caking effect, which is detrimental to the dispersion of the Si NPs and the electrode processing (Fig. S3c).

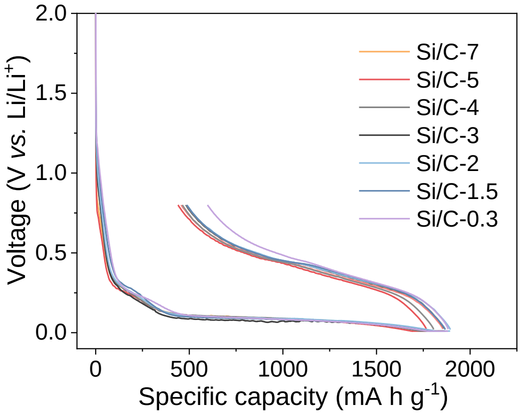

Fig. S4 The first charge/discharge capacities of Si/C composites.

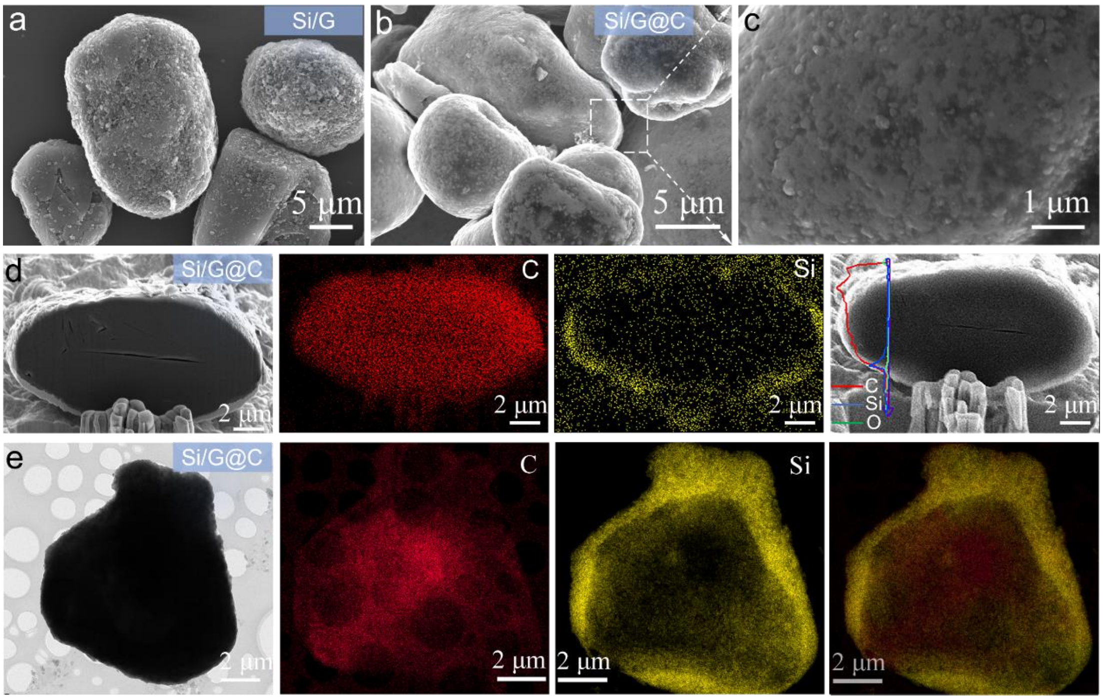

Fig. S5 (a) SEM of the Si/G composite. (b) SEM image of the Si/G@C composite. (c) The enlarged SEM image of the selected area marked by the white rectangle in Fig. S5b. (d) The cross-sectional image of Si/G@C composite processed by FIB, and corresponding EDS elemental maps of C and Si. (e) TEM image of the Si/G@C composite and corresponding EDS elemental maps of C and Si.

In stark contrast, the Si/G and Si/G@C composites exhibit aggregated Si NPs distributed on the smooth surface of Gr spheres, where the Si NPs may penetrate the scanty carbon coating layer (Fig. S5).

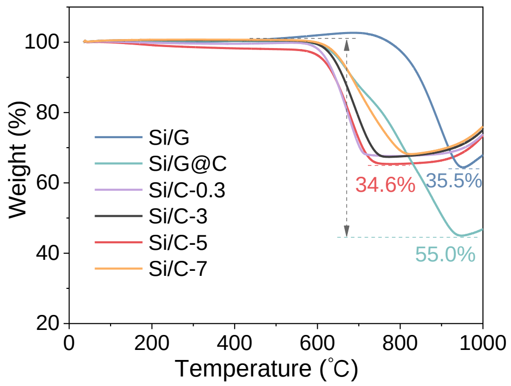

Fig. S6 TG profiles of the Si/G, Si/G@C, and Si/C composites.

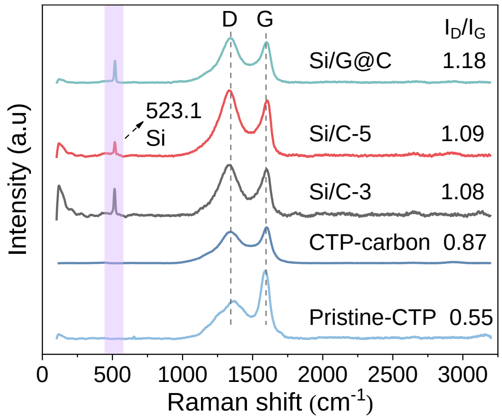

Fig. S7 Raman spectra of pristine-CTP, CTP pyrolysis carbon, Si/G/@C and Si/C composites.

The Raman spectra of composites were compared in Fig. S7. The peak at around 518 cm^{-1} is originated from crystal structure of Si. The D band at ~ 1350 cm^{-1} represents the defect-induced vibrations (disordered carbon). The G band at ~ 1580 cm^{-1} represents the sp2 bonded carbon atom vibration, corresponding to the first order scattering of the E2g mode. Noted that the I_D/I_G ratio of Si/G/@C (1.18) is higher than Si/C composites (1.08 ~ 1.09), indicating that the carbon coating treatment has introduced structural defects on the graphite scaffold.

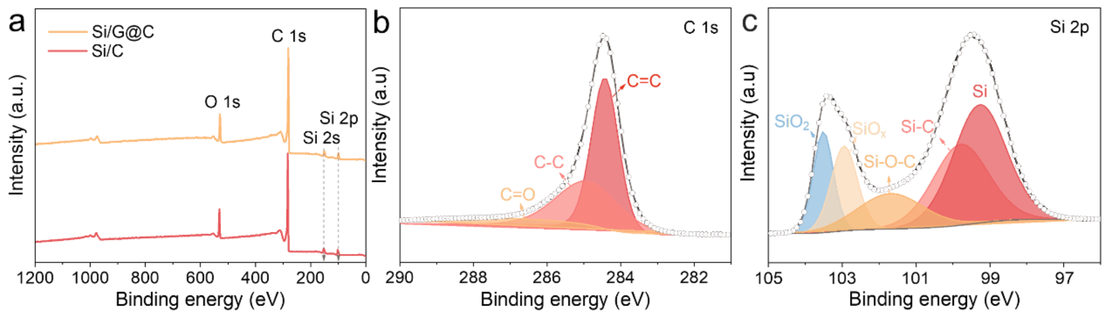

Fig. S8 (a) The full XPS spectrum, (b) High-resolution C 1s core-level spectrum, and (c) High-resolution Si 2p core-level spectrum of the Si/C-5 composite.

From the full survey spectrum of X-ray photoelectron spectroscopy (XPS) analysis in Fig. S8a, Si, C, and O elements can be detected in the Si/C-5 composite. Fig. S8b shows the high-resolution XPS spectrum of C 1s, which can be resolved into three individual peaks: C=C at the binding energy of 284.4 eV, C-C at 285.5 eV, and C=O at 286.8 eV. Fig. S8c shows the high-resolution XPS spectrum of Si 2p, which could be deconvoluted into four peaks. The distinct peak positioned at 98.9 eV is ascribed to the Si-Si bond; while the peak located at 103.5 eV is assigned to Si−O bond due to the partly interfacial oxidation of the Si NPs. In addition, two small peaks are observed at 100.5 eV and 101.6 eV corresponding to the Si-C bond and Si−O−C bond, respectively. The strong interfacial bonds (Si-C and Si−O−C) would not only enhance the interactions between graphite and Si NPs but also increase the electrical conductivity of the electrode materials.

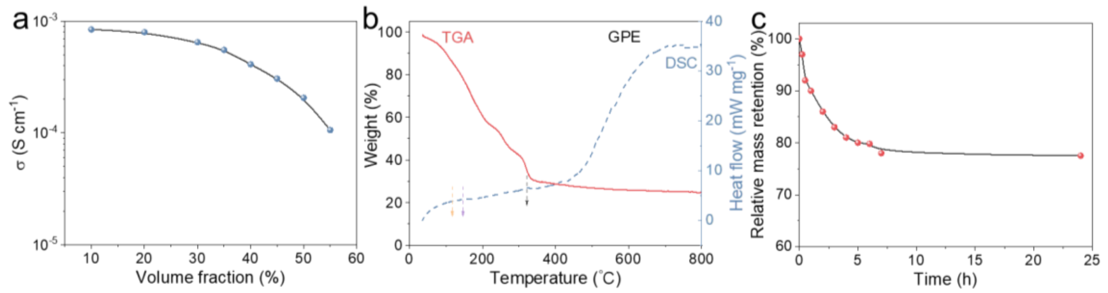

Fig. S9 (a) VC volume fraction dependent ionic conductivity of PVCM-GPE. (b) TG and DSC of PVCM-GPE with 40% VC. (c) Relative absorption radio of PVCM-GPE at 80 °C.

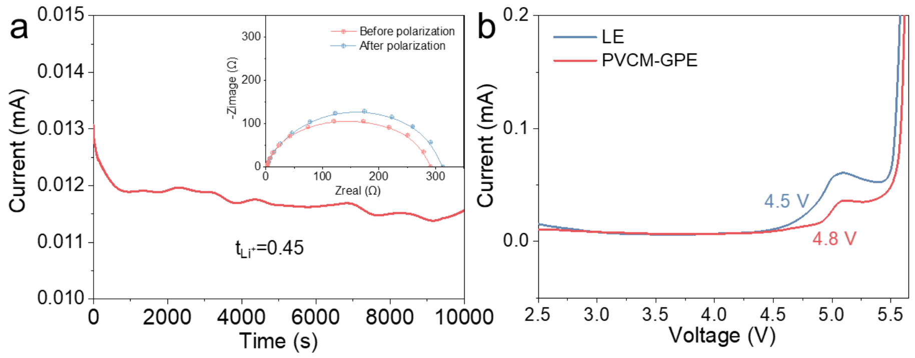

Fig. S10 (a) Polarization curves obtained by chronoamperometry and for the Li|PVCM-GPE|Li symmetrical cell. (b) Linear sweeping voltammetry curve of Li|PVCM-GPE|stainless-steel asymmetrical cell.

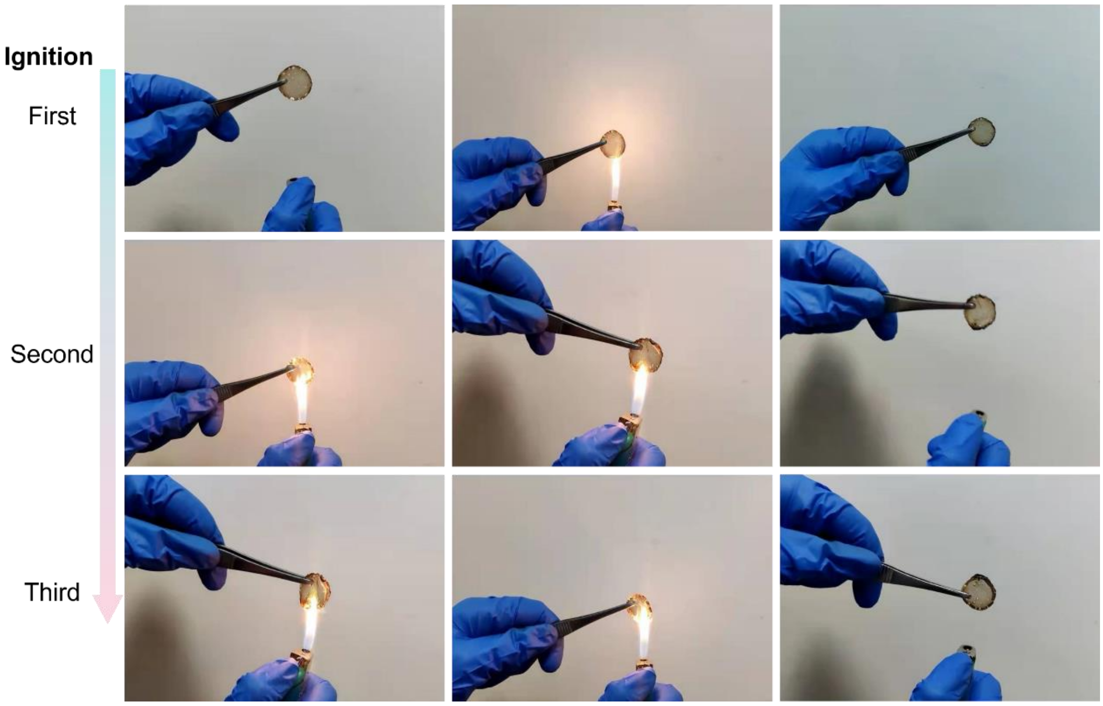

Fig. S11 PVCM-GPE was experienced combustion experiment for three times.

Table S1 Parameters used in the simulations.

Li/Si ratio / Parameters	Si	$Li_{12}Si_7$	Li_2Si	$Li_{15}Si_4$	$Li_{22}Si_5$
Young's modulus (Gpa)	115	90	75	65	40
Poisson's ratio	0.28	0.27	0.26	0.25	0.24

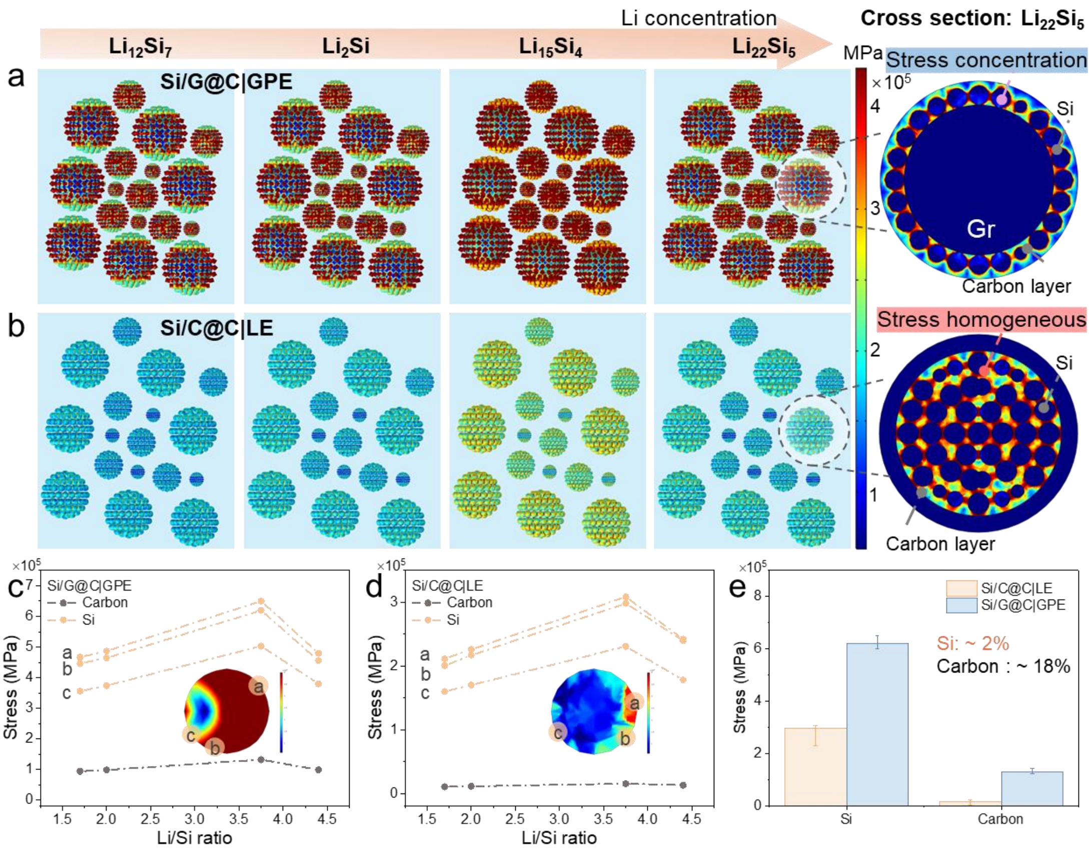

Fig. S12 Chemomechanical modeling of stress distribution during lithiation. Stress distribution modeling across the anodes for (a) Si/G@C|GPE and (b) Si/C@C|LE at different lithiation states, and stress distribution of the single composite particle at the deep lithiation state. Stress changes of Si NPs and carbon layer of (c) Si/G@C and (d) Si/C@C at 100% lithiation state. (e) Stress comparison of the Si and carbon layer species from two models.

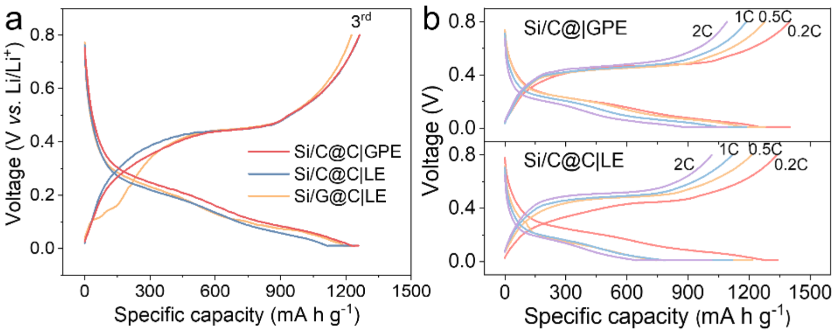

Fig. S13 (a) The voltage-capacity profiles of the third cycles at 0.5 C and (b) Rate performance of Si/C@C|GPE, Si/C@C|LE and Si/G@C|LE anodes.

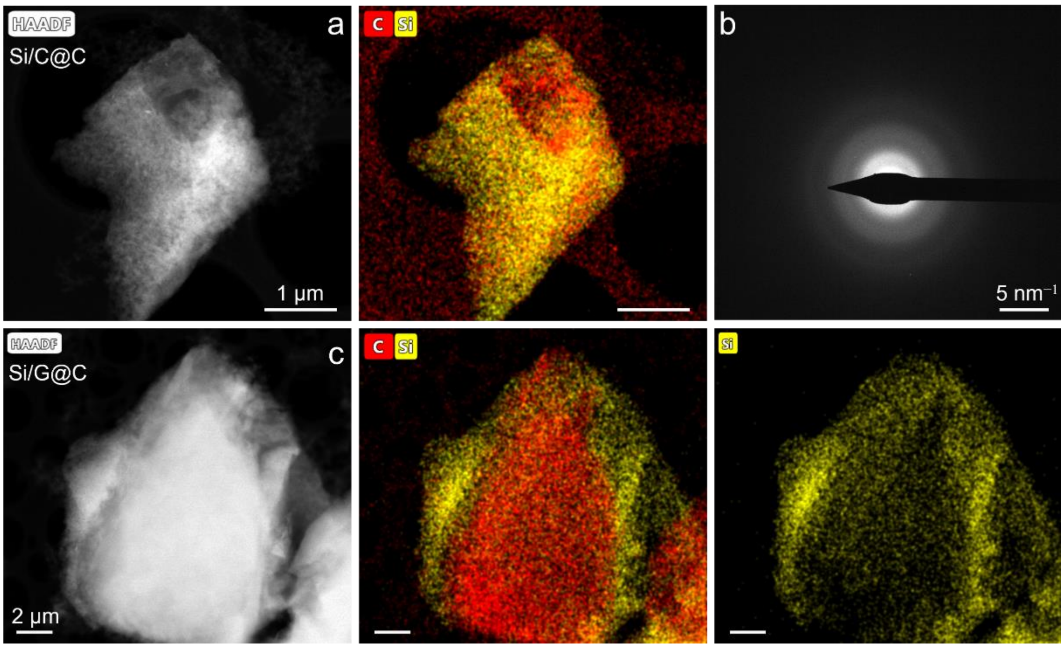

Fig. S14 TEM image of the (a) Si/C@C and (c) Si/G@C composites and corresponding EDS elemental maps of C and Si after 100 cycles. (b) SEAD pattern of the Si/C@C.

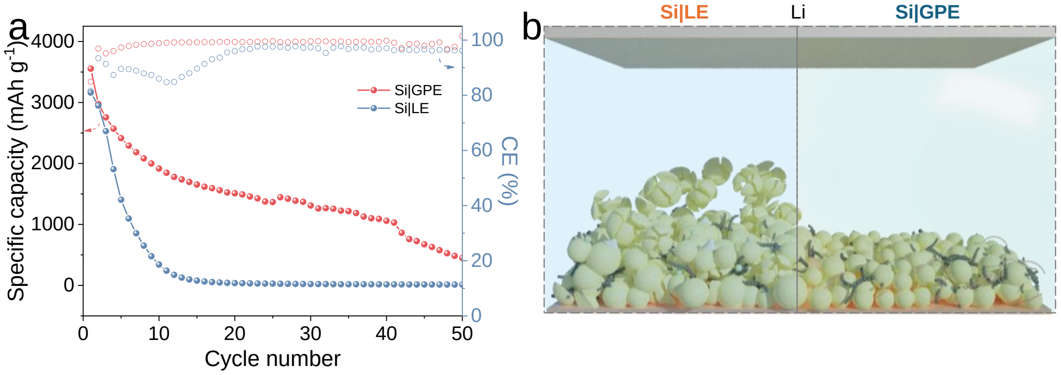

Fig. S15 (a) The cyclability values of the Si|GPE|Li and Si|LE|Li at 200 mA g^{-1}. (b) Schematic illustration of Si|GPE and Si|LE half-cell model.

Fig. S15 summarized the electrochemical performance of pure Si electrodes obtained in half-cells as paired with GPE or LE. The cycle behaviors of the Si|GPE and Si|LE anodes were compared at 200 mA g^{-1} with the similar areal capacity loading of ~ 2 mg cm^{-2} (Fig. S15a). The first discharge and charge capacities of Si|GPE were documented as 3554.6 mA h g^{-1} and 3016.6 mA h g^{-1}, rendering a satisfactory initial CE (ICE) of 84.9%. Meanwhile, Si|GPE renders better capacity retention (CR) of 44.1%

for 30 cycles and superior average CE value (98.9%) from the 3rd cycle onwards. For comparison, the Si|LE displayed the initial ICE value of 81.6% and 0.7% CR value for 30 cycles. Obviously, the difference in performance is determined by the different characteristics of the electrolyte. In the Si|GPE anode, PVCM-GPE encapsulation acts as a stress buffering layer to alleviate the volume expansion of silicon, which ensures the mechanical stability of the active particles during the lithiation, thus mitigating the loss of active material. Furthermore, this structural stability also avoids active Li+ depletion due to repeated generation of SEI (Fig. S15b).

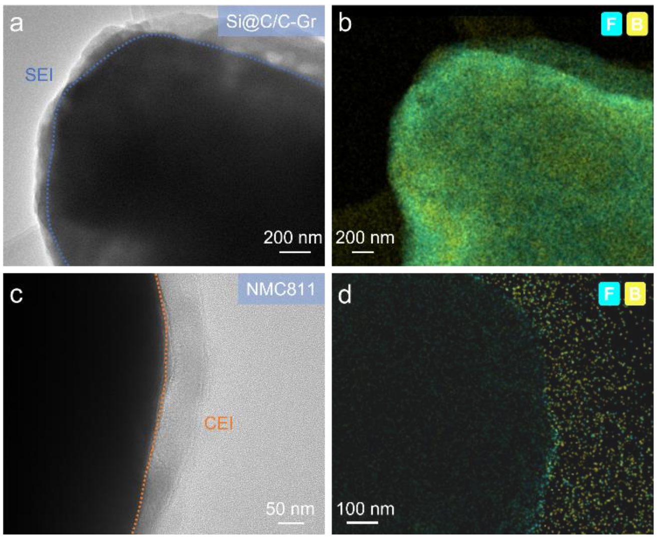

Fig. S16 (a) TEM images and (b) corresponding EDS elemental maps of F and B of Si/C@C-Gr in GPE after 100 cycles. (c) TEM images and (d) corresponding EDS elemental maps of F and B of NMC811 in GPE after 100 cycles.

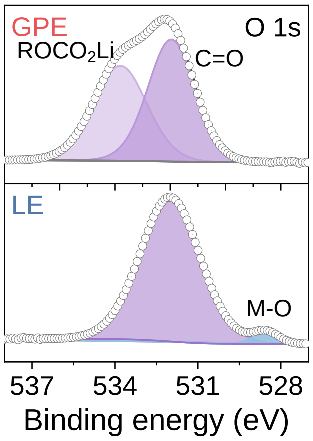

Fig. S17 O 1s XPS spectra of NMC811 cathode retrieved from Si/C@C-Gr|GPE|NMC811.

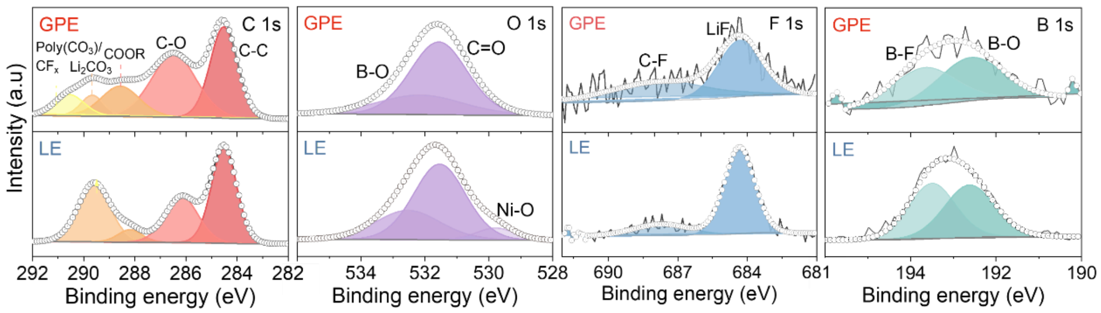

Fig. S18 C 1s, O 1s, F 1s, and B 1s XPS spectra of Si/C@C-Gr anode retrieved from Si/C@C-Gr|GPE|NMC811 full cell.

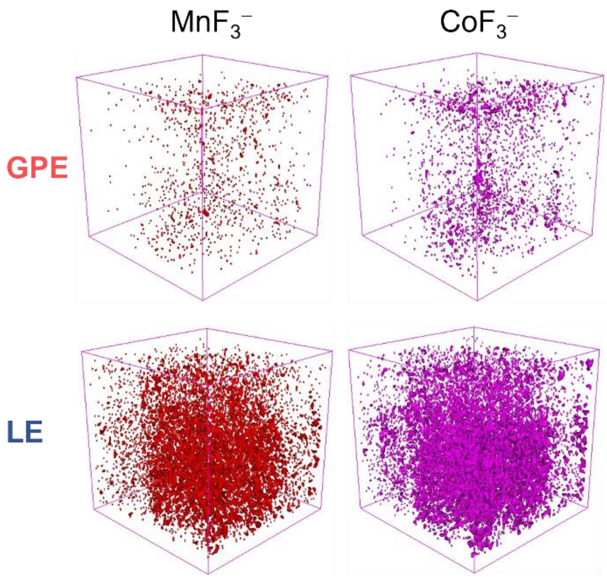

Fig. S19 TOF-SIMS 3D plots for MnF_3^- and CoF_3^-.

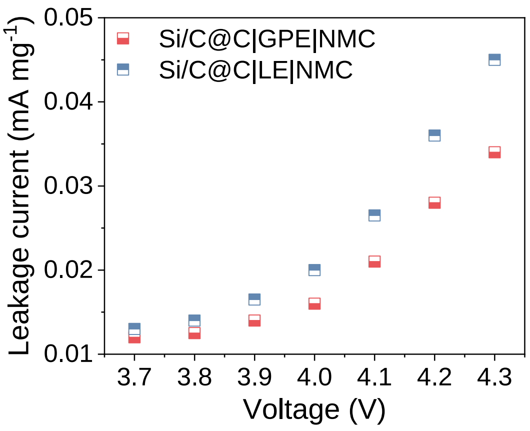

Fig. S20 Comparison of leakage current holding at different voltages for the Si/C@C-Gr 550|GPE|NMC and Si/C@C-Gr 550|GPE|NMC.

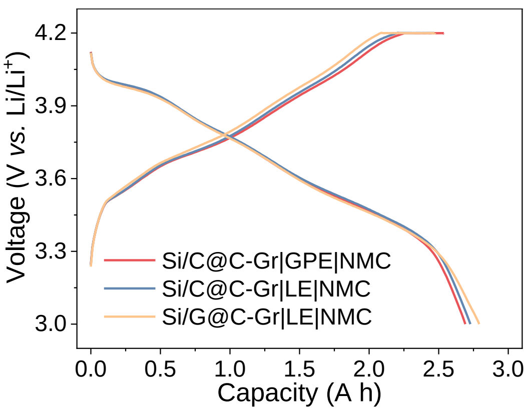

Fig. S21 The voltage-capacity profiles of the Si/C@C-Gr|GPE|NMC, Si/C@C-Gr 550|GPE|NMC, and Si/C@C-Gr 550|LE|NMC at 0.5 C

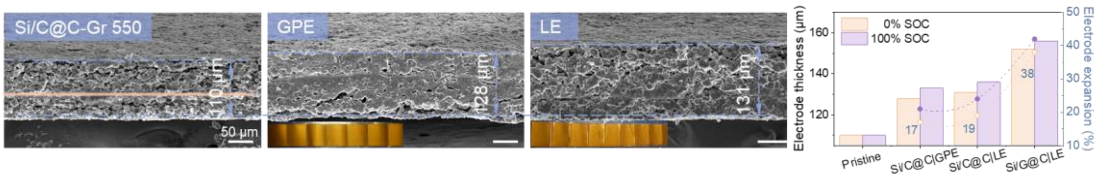

Fig. S22 Cross-sectional and top views FESEM images of the Si/C@C-Gr 550 electrode with GPE and LE before and after 100 cycles. Inset images are the optical photographs of the post-mortem Si/C@C-Gr 550|GPE and Si/C@C-Gr 550|LE electrodes, and summary of electrodes thickness variations.

From the photograph in Fig. S22, the Si/C@C-Gr 550|GPE electrode was flat, without any notable fractures on the surface. On the contrary, the mechanical strain of anode composite induces microcracks in Si/C@C-Gr 550|LE electrode, which also demonstrated 19% increase in the thickness at the lithiated state over 100 cycles. Worst of all, the Si/G@C electrode exhibits a 38% increase in thickness owing to the volume expansion of the aggregated Si without adequate reversed compression, leading to obvious electrode delamination upon cycling.

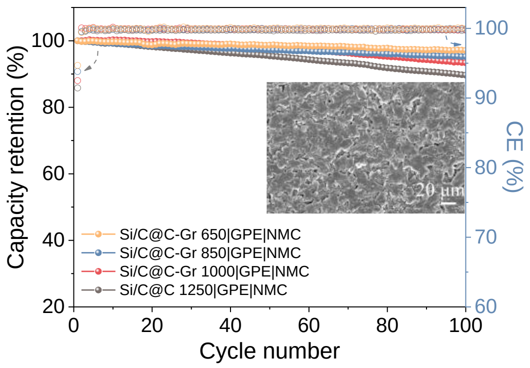

Fig. S23 CR of Si/C@C-Gr 650|GPE|NMC, Si/C@C-Gr 850|GPE|NMC, Si/C@C-Gr 1000|GPE|NMC, and Si/C@C 1250|GPE|NMC full cell prototypes at 0.5 C.

The energy density and power density were calculated by below equations:

Gravimetric energy density (W h kg^{-1})=$\frac{\text{capacity}}{\text{mass}}$×average voltage (V)

Gravimetric power density (W kg^{-1})

=gravimetric energy density (W h kg^{-1})× C rate (h^{-1})

Table S2 The calculation parameters of the main components and results of the gravimetric/volumetric energy density of the Si/C@C-Gr 550|GPE|NMC pouch cell.

Normal Capacity (Ah)			2.7		
Specific Anode Capacity (mA h g^{-1})	550	650	850	1000	1250
Output Voltage (V)	3.65	3.65	3.65	3.55	3.55
Area of Cathode (mm^2)			57*41*12 (layer)		
Area of Anode (mm^2)			59*43*13 (layer)		
Compaction Density of Cathode (g cm^{-3})			3.2		
Compaction Density of Anode (g cm^{-3})			1.6		
Areal Density of Cathode (g cm^{-2})			400		
Areal Density of Anode (g cm^{-2})	160	136	104	88	70
Electrolyte (g)	4.27	3.95	3.55	3.35	3.12
N/P Ratio			1.07		
Thickness of Al Foil (um)			12		
Thickness of Cu Foil (um)			8		
Thickness of Cathode Electrode (um)			137		
Thickness Anode Electrode (um)	110	93	73	63	52
Total Mass (g)	30.2	30.0	28.9	27.6	27.0
Energy Density (Wh Kg^{-1})	325.9	328.5	340.9	347.4	355.0

Table S3 The calculation results of the gravimetric energy density of the Si/C@C-Gr 550|GPE|NMC pouch cell.

C Rate	Gravimetric density (Wh kg^{-1})	Power density (W kg^{-1})
0.5	325.9	163
1	319.5	319.5
2	316.9	633.8
5	292.7	1463.5

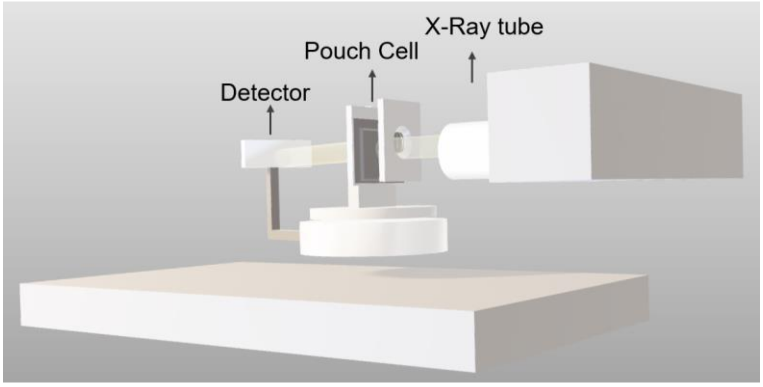

Fig. S24 Schematic illustration of operando XRD measurement of the Si-based anode||NMC full cell.

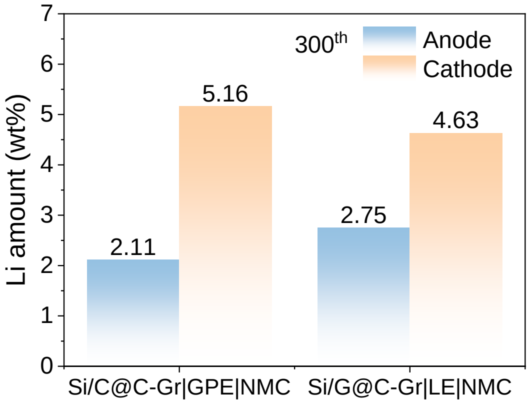

Fig. S25 Li quantification of the cycled electrodes by ICP-MS in Si/C@C-Gr 1000|GPE|NMC811 and Si/G@C-Gr|LE|NMC811 prototypes.
